# Attitudes to and Experiences of Physical Activity among Migrant Women from Former Yugoslavia –– a qualitative interview study about physical activity and its beneficial effect on heart health, in Malmö, Sweden

**DOI:** 10.3934/publichealth.2015.2.194

**Published:** 2015-05-08

**Authors:** Elin Sandström, Ingrid Bolmsjö, Ellis Janzon

**Affiliations:** 1 The national Swedish organization for consumers, Stockholm, Sweden; 2 Health and Society, Faculty of Health and Society, Malmö University, Malmö, Sweden

**Keywords:** culture, heart disease, migrants, overweight, physical activity, women

## Abstract

**Background:**

Many risk factors for heart disease can be reduced by lifestyle modifications such as physical activity, but the attitude to and the knowledge about the beneficial effect of physical activity vary among the population. Migrant women are reported to have a higher BMI and to be less physically active than the Swedish-born women. In order to motivate them to participate in physical activity it is necessary to understand that they are not a homogenous group, and thus their knowledge about, needs for, and attitude to physical activity have to be examined. Aim: The aim of the study was to explore structural and individual factors working either as barriers against or as motivation for a change towards higher levels of physical activity and a healthy lifestyle. Furthermore, the aim was to investigate if the migration had changed the women's level of physical activity and what would be required to increase it.

**Method:**

Seven women from Bosnia living in Malmö, Sweden, were interviewed by means of a semi-structured interview guide. The data was analyzed using Burnard's content analysis method.

**Results:**

The findings were presented in two categories, namely, “barriers against physical activity” and “motivational factors for physical activity”. With regard to the category “barriers against physical activity”, the move to Sweden had led to losses and shifts in lifestyles for the women. The greatest lifestyle changes were reported among women who had moved from rural areas in Bosnia to urban areas in Sweden. They found it troublesome to reach the same activity level in Sweden and expressed a greater need to do so. Earlier negative experiences or no experiences at all, of performing physical activity, as well as the winter climate, were seen as obstacles to being active. All the women prioritized family, work, school, and club activities above physical activity. With respect to the category “motivational factors for physical activity”, it was found that physical activity could help improve their mental balance, and the women also considered the possibility of losing weight.

**Conclusion:**

The study showed that although these migrant women had difficulties finding appropriate and realistic physical activities, and prioritized family activities, they desired to be more physically active, even if the climate was seen as a hindrance. They also reported that physical activity could be a means to achieve better mental health as well as weight loss. Politicians ought to allocate funding, and public health worker to focus more on and enable this high risk group of immigrant women to become more physical active. They should also be informed about their increased risk of myocardial infarction. This, to stimulate increased physical activity among them and in ought to be in co-working with their own immigrant organization.

## 1. Introduction

In Sweden, cardiovascular disease (CVD) and mortality have decreased during the last twenty years due to increasingly effective risk factor management and more effective post cardiac hospital treatment. Still, the risk of having a myocardial infarction (MI) has not decreased in all groups, e.g. not among middle-aged and/or migrant women, where it is stable. However, the incidence is still lower than in men. Sweden has had a large migrant influx during the last ten years, and some cultural habits and culture-associated lifestyle factors, such as diet and physical activities, change slower in all population groups, and consequently also among migrant women, and this might affect the incidence [1]. A recent study made by this group confirmed what has also been shown by others, namely, that migrant women have a slightly higher BMI and are a little less physically active than their Swedish counterparts [2],[3]. This gives them a higher risk for other diseases as well, such as diabetes, higher blood pressure, and higher blood-lipid levels, risk factors that can be modified by physical activity [4]. Another study showed that the migrant women belonging to a lower socio-economic level in Sweden were more vulnerable to both stroke and MI [3]. Some groups of migrant women in Sweden might be especially vulnerable, due to involuntary refugee migration, and to becoming isolated and getting a lower socio-economic status in their new country. Some of them also have a cultural preference for a larger body size [5],[6] and some are unaware of the benefits of physical activity [4]. As physical activity can improve both physical and mental health [6], it is important to increase the interest in physical activity in the Swedish population, just as goal number nine of the National Goals for Public Health stipulates, and to emphasize that this applies to the whole population [7].

As migrant women seem to have a higher BMI and are less physically active [2],[3],[8],[9] than Swedish-born women, they need to be given extra attention regarding their attitudes to physical activity, and they need increased knowledge about the risk factors for heart disease and about the benefits of physical activity. Still, this might differ between migrant groups. As it had emerged that migrants from former Yugoslavia had an increased risk of developing myocardial infarction, even at a younger age (≤ 40 years), compared to others [10], it was found interesting to further explore the perceptions in this group, since many risk factors for MI are preventable [11],[12].

In this study, we are discussing leisure-time physical activity, and in Sweden about 65 percent of all men and women, between 16 and 29 years of age, follow the Swedish Public Health recommendation of at least 30 minutes daily physical activity. The group of women interviewed for this study are middle-aged, however, physical activities normally decrease with increasing age [7a]–[b] and persons born outside Europe are less physically active than Swedish-born persons. Only 33 percent of women born outside Scandinavia are physically active compared with Swedish-born women (52%). Migrant women from countries outside Europe are, thus, more sedentary than Swedish women [4], [6]. One strategy that is becoming more and more common in Sweden is the use of *Prescription of Physical Activity on Recipe* (FaR), a part of the strategies of the National Public Health Institute in their effort to increase physical activity among the population [7a]–[b]. This strategy is now a task for the primary health care organization and the prescriptions can be dispensed by both doctors and nurses, and also by other caregivers, and are targeted at inactive patients at no cost for them.

The interactions of attitudes and beliefs related to physical activity across groups, cultures, and countries, are complex. A study performed across Europe showed that there are wide variations in whether body weight or physical activity was perceived as influential on health. Overall, smoking, food, and stress were all perceived as the most important influences on health [11]. Situational factors, such as migration and health status, are influential on women's own attitudes towards physical activity. The cultural heritage that migrant women carry with them with regard to body image, what physical activity is, and who should conduct it, may differ widely [5],[6]. For some migrant women, physical activity was perceived as being only for the youth, and the dress code and/or the place for the conduct of physical activity were not always acceptable [5]. Furthermore, many migrant women who would want to participate in physical activity classes, wished for group activities being arranged only for women [5],[8].

However, the migrant women's reasons for being physically inactive might also differ, as well as their needs to make leisure-time physical activity possible. Many migrant women are taking more family responsibility than Swedish-born women and are thereby more tied to their homes [4],[8],[11], but there can be other reasons for being inactive.

The aim of the study was to explore structural and individual factors working either as barriers against or as motivation for a change towards higher levels of physical activity and a healthy lifestyle. Furthermore, the aim was to investigate if the migration had changed the women's level of physical activity and what would be required to increase it, if needed.

## 2. Method

### 2.1 The interviews

The interviews were conducted in 2012, in Malmö, Sweden. All participants understood and spoke Swedish after many years in the country.

In order to access the women's perspective on physical activity and body ideal, individual face-to-face interviews were performed in the homes of the respondents. The interviews were semi-structured, following a thematically structured interview guide, with questions regarding general information about the women, perception about health before now, what the move had meant for their health behavior, their own body image, motivation for leisure-time physical activity, and obstacles to and needs for being able to perform physical activity. The questions were open-ended [13]. The interview guide was constantly revised and updated with further questions, when necessary. The interviews lasted 30 to 60 minutes and were recorded and transcribed verbatim and afterwards translated into English. The interviews were tape-recorded in order not to lose important information and the respondents gave their permission to this. Afterwards they were checked for accuracy by the group of scientists.

### 2.2. Respondents

In order to achieve a purposive sample and get contact with women from Bosnia living in Malmö, Sweden, the snowball technique was used, based on two different first contacts from the Bosnia Immigrant Organization, in Malmö. The recruitment of respondents took time and many attempts were made to include more women with the same background that had migrated during the same period and were all born in Bosnia during the early 1990s. At every interview, the respondent was asked if she knew and would recommend other women with the same background, and if so would she kindly request that person to take contact in case of interest to participate. At last seven women were included, even if more respondents had been wished for. In purposive sampling, subjects are selected because of their ability to share some important information and because of their relevance to the studied topic. Together the subjects, ought to, represent the variety within the source population, and we wished for women of different age, but with the same immigration background [13]–[15].

Included were to be women, immigrated to Sweden from Bosnia after 1992, who had been more than ten years in Sweden. The seven women that participated in this study were born between 1953 and 1990 in Bosnia. They came to Sweden during 1992–1993 due to the war conflict, which means that two of the women had lived most of their lives in Sweden. Only one was under 30 years of age, six women were 40–60 years. They did not suffer from any diseases and they all stated that they knew their weight and had increased it after the move to Sweden (Table 1).

**Table 1.** Seven immigrant women from Bosnia 1992-1993.

Number of women	7
**Age group**	
**< 30**	1
**> 40–60**	6
**Education**	
**University**	1
**High school**	5
**Practical education**	1
**Employed**	7
**None smoker**	5
**Occasional smoker**	1
**Daily smoker**	1

### 2.3. Analysis

A qualitative analyzing process guided by grounded theory offers a systematic way of transforming collected data into detailed and re-contextualized information [14]. Burnard offers a method that is based on the ideas of, among others, Glaser and Strauss (grounded theory) and that stays close to the original material but allows categories that make sense of the data to be generated [16]. As soon as the interviews were transcribed, the analysis and the coding process started. Burnard's twelve-stage analyzing process started immediately with memo writing after the first interview [16]. Open coding of the transcripts was used, which means that codes were not decided on beforehand but particular key words that captured the essence of the area were selected, while the transcripts were read line by line. Saturation continued as each interview was analyzed and coded. The codes were then compared and grouped into broader categories. All items belonging to each code were collected and served as a description of each category. As the process continued, a main category that could explain and be linked to the broader categories was developed. The writing process started when all transcripts had been coded and the categories filled with information. The data was collected by one of the group scientists, but data was analyzed and coded by the whole group after each member had read the interviews.

### 2.4. Ethical considerations

An effort has been made to make sure that confidentiality was secured throughout the research process. The participants have been de-identified and no real names are used. The Ethical Board of Scania and The Ethical Board of Malmö University approved the study. All respondents gave informed signed consent.

### 2.5. Trustworthiness, validity, and transferability

Throughout the research process the following aspects have been taken into consideration: the trustworthiness of the study, and how the research question, the data collection, the nature of the studied phenomena, and the analyzing method harmonized [17].

During the interview situation the information was validated through follow-up questions and questions intended to make sure that the answers had been correctly understood. The interview guide with open-ended questions guided the interviews, and leading questions were avoided. The analysis process started without pre-conceived codes, and as the process continued, comparisons of codes within and between categories were made in order to check consistency and accuracy. Some categories were rejected, since they did not sufficiently contribute to the emerging main process. The coding and analyzing process was conducted by the group of scientists involved

The transcriptions of the interviews were not read by the respondents. They were offered to read them, which would have been a strength for the study, but they showed no interest, and we did not insist.

## 3. Results

As the aim of the study was to explore structural and individual factors working either as barriers against or as motivation for a change towards higher levels of physical activity and a healthy lifestyle, the findings have been presented through the two explanatory categories *“barriers against physical activity”* and “motivational factors for physical activity”. The two categories influenced the core process that evolved into *“attitude towards physical activity”.*

### 3.1. Factors working as barriers against physical activity and a healthy lifestyle

#### 3.1.1. Self-awareness and lifestyle

Whether the women considered themselves to have a physically active lifestyle or not varied between individuals. They reported that the level of physical activity varied in a lifetime perspective. To be or to become physically active could, for them, be described as a process, a process influenced by many factors. The involuntary move to Sweden due to the conflicts in former Yugoslavia during the early nineties had led to losses and shifts in their lifestyle. The greatest lifestyle changes were reported by the women who moved from rural areas in Bosnia to urban areas in Sweden. The women who lived in urban areas in Bosnia and who continued to live in urban areas in Sweden had not experienced any substantial lifestyle changes. On the other hand, the women from rural areas reported that they had lost the natural physical activity they had before through their former country life. They found it troublesome to reach the same activity level in Sweden, and experienced a greater need of more consciously planned activities than before the move.

“We did not learn to go somewhere to exercise and set aside time for exercise. It happened at the same time as you worked /…/.It was exercise for free; we walked up and down the hills and there was no need for exercise. I was as thin as a stick.” (Respondent 1, aged 60)

#### 3.1.2. Lack of experience of physical activity

The ability to plan and realize physical activities requires both personal and physical resources. Not having any earlier experience of planned physical activity, such as experience of workout in a gym or being a member of a sports association, was mentioned as a barrier to starting with some kind of planned activity:

“I have not grown up with the idea of going somewhere to exercise /…/ so I don't have any experience of that from Bosnia. /…/ I don't think that anyone can convince me to start with some kind of physical activity where some instructor shouts at me. /…/ I don't want to do that now, I want to do it when I feel like it. I don't know why it is like that but probably it's because I'm not used to it and I have not grown up with anything like that /.../.” (Respondent 2, aged 50)

Experience from physical education in school was also influential with regard to the attitude towards physical activity. The women, who reported bad experiences from childhood or had little interest as children in physical activities, said that the negative attitude had followed them into adulthood. That attitude had influenced a lifestyle with limited planned physical activity, but some women reported that new positive experiences of physical activity could change that attitude.

“It is actually the case that I have never thought of it [physical activity] as fun.” (Respondent 4, aged 50)

#### 3.1.3. Climate and environmental factors

An aspect that appeared during the interviews was the adaptation to the Swedish climate. Darkness, cold weather, rain, and snow were all factors that influenced the willingness and the possibility to move outside during the winter months and days with bad weather. For those women who did not practice any kind of indoor physical activity, the climate was influential on how much physical activity they performed during periods of bad weather. The women recounted that it had been a process to learn how to dress when it is below zero and snowy.

“Both climate and periods of darkness have an effect on me, because during the summer I am active. /…/ we can go for a walk at nine o'clock in the evening but when it is winter and it is six o'clock I don't want to go out, I don't have any motivation /…/. So yes, the climate influences me, it does.” (Respondent 2, aged 50)

#### 3.1.4. Capacity to prioritize

To prioritize planned physical activities was not seen as a simple task for the women. Other interests and duties, such as family, work, school, and club activities, were prioritized above physical activity. One of the women emphasized the difficulty of choosing between different activities in life that were valued as important.

“Time is not always sufficient. Other activities take over, school, for example, but even if school is more important in one way, if you think that health should be prioritized, then you don't really know.” (Respondent 6, aged 20)

Several of the women did not consider planned physical activity as an interesting and meaningful leisure-time activity. A physically inactive lifestyle with activities such as watching television and socializing with the family was for one of the women considered more enjoyable than planned physical 3activity. All the women said that they were ultimately responsible themselves for being physically active. Laziness and lack of energy were mentioned as two personal barriers against planned physical activity. One of the women who had tried to change her lifestyle and become more physically active said that:

“It is difficult to find an activity that you can accept.” (Respondent 3, aged 50)

Having a goal that you want to achieve was mentioned as a good motivation by one of the women. But once the goal of losing weight was achieved, this woman had difficulties continuing being physically active and keeping up the interest; she thought that she could now take it easier and thus she fell back into old routines.

“/…/ now my goal is achieved, so I can take it a little bit easier.” (Respondent 3, aged 50)

Overall the women reported that it was difficult to stay physically active over time. Lifestyle changes were perceived as difficult and demanding. Speaking from experience, one of the women said that:

“At the outset you want to change everything drastically, but it doesn't work /…/. I have to do everything in small steps, for it not to be too big a change.” (Respondent 5, aged 40)

#### 3.1.5. Health and mental health

Several of the women mentioned the importance of physical activity for their mental health. They experienced that physical activity contributed positively to their mental health, but at the same time their mental health problems could act as barriers against physical activity sometimes. Stress, anxiety, sleeping disorders, and tiredness all decreased the motivation to exercise.

“/…/ because I know that I sleep very badly and when I wake up on the day when I'm going to ‘work out ‘my body won't have the strength for it, my body resists. But if I sleep, then I will have more strength.” (Respondent 6, aged 20)

#### 3.1.6. Economy and price of activities

The price of activities in, among others, sports centers was crucial. Economic conditions influenced if the women could afford to be physically active in a sports center. Some of the respondents reported that they could not afford a membership, or that they had not been able to afford it earlier.

“/…/ most of the time it has been the price, because I did not work during junior high and high school, so I had to ask my mother if I could have money for the gym. And our economic situation was not very good, so then it was no. That's also why I was so negative towards gyms and exercise overall.” (Respondent 6, aged 20)

Still, some of the women exercised regularly through their workplace, since they were offered the opportunity to exercise on work time and got subsidized for physical activity costs, but not all who were offered that possibility used it. For these women, the price was not the barrier; instead they prioritized other activities.

### 3.2. Motivation for a change towards higher levels of physical activity and a healthy lifestyle

#### 3.2.1. Outcome, demand, health, and mental health

The experience of physical activities improving health was a motivating factor for continued activities for those of the women who were active or who had been active earlier:

“/…/ but now I believe that it is very important, because I notice that I feel much better when I'm active.” (Respondent 6, age 20)

The women mentioned that it was not only the physical aspects, like fewer colds or increased strength that motivated physical activity, but also the mental health gains. They had experienced increased concentration, happiness, and improvements in mood, when they were physically active. The joy of experiencing the possibility of achieving goals and of seeing progress in their training was also mentioned as a motivational factor.

“/…/ oh it makes me happy! It is really tiresome during workout but I become so positive then, so I just keep on, keep on because I know that it makes me feel good and that's why I do it. I ‘work out’ to feel good, I don't ‘work out’ to look good for someone else but to feel good, simply /…/. Where I found motivation for it? In myself. I push myself. You have to manage it! But also because my concentration improves. That's also why I work out now, because I have felt that my concentration on my studies has not been so good, so I thought that I'd try with some workout and be a bit more active and see if it helps and it has become better, so it pushes me even more /…/.” (Respondent 6, aged 20)

How much planned physical activity the women experienced as sufficient varied? Most of them thought that they needed more regular exercise than they got at the moment, but it depended on how much physical activity they got throughout a day. If they walked or biked to work earlier but changed to going by car and hence to a more inactive lifestyle, that meant an increased need for planned physical activities. When the women talked about planned physical activities it was mainly connected with the need of weight loss. Less focus was placed on other health gains or positive outcomes of a more active lifestyle. None of the women believed that they had had any signs of CVDs, but they were not sure since they did not know the symptoms. They were not especially afraid of CVDs, either, or of falling ill; they were more afraid of becoming dependent on someone else due to illness.

#### 3.2.2. Meaningfulness, locus of control, and inner motivation

For some women, the main goal of being physically active did not necessarily have to be weight loss or improved fitness. Exercise and weight loss would, preferably, come as positive “side effects” of activities that were performed for the purpose of having fun. The women wanted the activity to have some kind of meaningfulness and a goal. One of the women expressed that using a stationary bike for 30 minutes, or doing weight training, did not feel like a meaningful or joyful activity. To walk with friends, however, could be meaningful:

“Something that can feel a bit more fun. Like those walks, for example. You socialize and talk about things and it feels a bit meaningful and then you have exercised as well.”(Respondent 3, aged 50)

A recurring comment among the women was that others could both encourage and influence them, but it was the women themselves who were responsible for their own training. They wanted to be in control of how and when to exercise. Whether they preferred to exercise alone or together with someone varied very much between the individuals.

“Sometimes I feel trapped: we are ‘going to ‘work out’ now, now it's time for group activity and such. Then I go and exercise by myself. I don't want any rush after work /…/. But I would not have been as good a year ago. I have now realized that these group activities make me go even at times when I don't feel like I have the energy.” (Respondent 5, aged 40)

#### 3.2.3. Lifestyle, body ideal, and diet

To follow a diet and/or have the goal of losing weight were mentioned as motivational factors for physical activity for some of the women. To take the step from wanting to lose weight to actually doing something about it was considered very difficult. One barrier was the experience of not being in control over the intake of food. Some of the women said that their diets had changed radically since they came to Sweden. Due to lack of time, prices, limited supply, and differing desires on the part of family members, some of the women felt that they could not eat the type of traditional food they used to eat in their homeland, Bosnia. Some of the women considered that the “Swedish food” they now consumed was less healthy and a contributor to their overweight. Several of the women had gained weight during their first years in Sweden and they said that their bodies had undergone major shifts.

#### 3.2.4. Earlier experiences of physical activity

The women in the study who had earlier experiences of planned physical activity were more positive to physical activity than those of the women who had not been active earlier. Experiences of regular activities contributed to knowledge about positive outcomes, how it was felt in the body, how to tackle barriers, and what they could expect when preparing and planning for new practices. Hence, how physically active one has been earlier in life seems to be important for one's activity levels as an adult.

#### 3.2.5. Physical Activity on Receipt (FaR)

None of the women in the study had had much contact with the healthcare or could remember that they had ever had any lifestyle advice during a healthcare visit.

“just never heard about it” (FaR) I never had any lifestyle advice at the health care center” (Respondent 3, aged 50)

“Oh, I see! But it was never offered to me, but if it had been, I might have been interested in trying.” ((Respondent 5, aged 40)

## 4. Discussion

The aim of the study was to explore structural and individual factors working either as barriers against or as motivation for a change towards higher levels of and a healthy lifestyle. Furthermore, the aim was to investigate if the migration from Bosnia to Malmö, Sweden had changed the women's level of physical activity and what would be required to increase it, if needed.

Only a few of the respondents were physical active, some had been active now and then in the time passing after their move to Sweden. We found that a physically active lifestyle was a challenging task for the respondents but still regarded as important for a healthy lifestyle. No selection was made to include any Swedish middle-aged women since there was no intention to compare the groups. The interest in exploring obstacles and attitudes to leisure-time physical activity among different groups of migrant women is to increase physical activity and thereby decrease the risk of heart disease [5].

It is important to state that the results in this study are limited to the women who were included here and hence might not be attributed to all other women. We would also like to state that even if the sample of women in this study was smaller than wished for, the interviews were rich of data and fulfilled the aim. However, we could have insisted in letting the respondents read the written transcriptions of the interviews, as verification. Still, the transferability to other contexts has been considered by relating the results to existing theories and models in the area.

### 4.1. Factors working as barriers against physical activity and a healthy lifestyle

Whether the women in this study considered themselves to have a physically active lifestyle varied between individuals, and they found that the level of activity also varied in a lifelong perspective. The attitude towards physical activity *had* been changed over time. Several of the women reported that they had been physically active in their homeland (Bosnia) or during periods in Sweden but that, among other things, the relocation to Sweden had changed the conditions for a physically active lifestyle.

One of the skills belonging to self-efficacy is the ability to plan one's time. Several of the women in this study said that they found it difficult to prioritize between different activities and chores and to make time for physical activity. Earlier research has reported that trying to push physical activity into an already tight schedule can instead lead to negative feelings towards being physically active and hence have the opposite effect, leading to less activity [18].

Commitments, such as work, family, and club activities, were perceived as obstacles, to planned physical activity, among the interviewed women. By strengthening a person's self-efficacy, the feeling of control over their environment can be enhanced, so that the person can plan and prioritize between different activities in relation to her life-goals. Physical activity is also more likely to be continued long term if the goal of the activity is related to other things in life than body shape, toning, or losing weight [16],[18],[19]. As expressed by the women, other aspects were more important, especially their family. Some women reported that if they had to choose between physical activity and family, family came first. This was a significant message, since these women were not only migrants but also refugees due to the earlier war conflict in their homeland. They might have experienced loss of family members, so to keep close to family and friends was important to them. This fact might be crucial to recognize and to take into account when planning public health measures, as many people today migrate to other countries due to conflicts in their home countries. There are different ethnic groups of migrants in Sweden, as elsewhere, and most of them have their own organizations. It is important and recommended that public health measures are communicated through and together with the migrants' own organizations in order to be successful. Even if some of the women expressed that they wanted more time for themselves, to be able to spend more time with friends or “work out”, this could be arranged within their migrant organization. One suggestion could be to focus primarily on their sense of well-being, using stress reduction methods, rather than on “high tempo enjoyment” as intrinsic motives for physical activity among midlife women [20],[21].

Information that recurred in the results was earlier experience of physical activity. It seems important for one's activity level as an adult to have had positive experiences of physical activity earlier in life. Several of the women in this study had no earlier experience of physical activity in their childhood, in school education, or if so failed attempts at being physically active later in life that influenced their attitude negatively towards being active today. This shows how important it is to encourage children to become physically active early on in order for them to remain physically active as adults. Furthermore, it shows that social relations, that is, parents, teachers, and friends that can inspire physical activity, are extremely important during childhood, just as supportive environments (workplaces) are very important later in life. This is also supported in an earlier study performed in Sweden, showing that earlier experiences of exercise among migrant women in Sweden were found to be more important than how long they had lived in Sweden or their language skills [22].

Lack of accessibility of spaces for physical activity in the neighborhood as well as the climate were mentioned as obstacles to physical activity outdoors during the winter and on days with bad weather. Hence, the accessibility of economically reasonable indoor facilities needs to be increased. None of the women believed that there was a lack of facilities; the problem was to find an activity that felt suitable or was affordable.

### 4.2. Factors that motivated, or could be motivational for, increased physical activity for a healthy lifestyle

It is most important to create supportive environments and find innovative solutions that encourage physical activity. The women experienced a loss of physical activity due to changed living conditions and felt that they had become more sedentary. The labor market has changed more and more, so that we get less and less of our daily activity during working hours [7a]–[b]. It was difficult for the women to find time for planned physical activity in their lives after the move. Some of the women got some benefits through their work, such as subsidized costs of activities or the possibility to exercise during working hours. For the women who worked in a workplace where there was a culture of exercise, the opportunity to exercise during working hours was seen as positive and valuable. It motivated them to be more active also in their spare time and to use the subsidies offered for the costs of physical activities. To encourage a positive exercise culture in the workplace and set aside time for physical activity during working hours could be an important strategy to increase the physical activity level. The high price of different activities was another factor mentioned by some women as a reason for inactivity. Subsidies on costs of memberships in health centers or associations could make it economically possible for more women to use these facilities. However, only full-time employed persons are included in these benefit programs. Another strategy could be to encourage workout centers to offer reduced prices to migrant women as well as to senior citizens and students.

All the women in this study said that they had a need for more and regular physical activity physical activity, and one motive was a desired weight loss. Before initiating, for example, leisure-time physical activity, it is mainly external bodily changes, such as weight loss, that motivate the start of a health-behavior change process and it is mainly internal factors, such as perceived satisfaction, that motivate continued activity [18]. This was seen in the stories of the women. For the women who contemplated a change, motivation focused mainly around perceived overweight and weight loss. From what the women report, it seems as if the goal of physical activity changes from being predominantly related to weight loss to being more related to the positive feelings and outcomes of physical activity over time.

### 4.3. Physical activity on prescription (FaR ) could be motivational and ought to be suggested

None of the women in the study had heard of physical activity on prescription, that is, the possibility for doctors and nurses in Sweden to prescribe physical activity. After some information about the concept, they were all positive to the idea of a prescription, if needed. The possibility to get an alternative form of treatment instead of traditional advice, or medical treatment, was seen as something positive. Continued work to spread the knowledge about physical activity on prescription is important [7a]–[b]. None of the women in the study had much contact with the healthcare or could, surprisingly enough, remember that they had ever had any lifestyle advice during a healthcare visit. Healthcare has an important role in the preventive work, especially with the possibility for healthcare workers to reach people and to inform about and prescribe physical activity, and not only as exercise training; they can also inform about the fact that healthcare centers teach different relaxation techniques, something that there seems, according to this study, to be a need for. It is an easy and a popular public health task to engage young people in physical activity, since they mostly have a natural interest in being active. It is, however, more of a challenge to activate vulnerable elderly women, such as those who have low socio-economic status or are migrants. This demanding challenge needs to be taken seriously by the primary healthcare as well as by the public health workers, and not least by the politicians allocating resources, and meeting the challenge will be rewarding in the long run due to a decreasing incidence of disease as well as decreasing healthcare costs. Furthermore, it is important to spread the news that physical activity can be more than trendy high tempo/music exercise, and that it can also be more calming training, like yoga, or training forms more directed to the elderly population, who might need it for disease prevention. For the prevention of cardiovascular disease, walking is just as good as “strenuous” exercise [24].

### 4.4. Supportive environments

The environment in which we live influences how physically active we are. To live in a neighborhood with safe and attractive in- and outdoor facilities for physical activity, with bike lanes, pedestrian precincts, and recreational areas, increases the levels of activity. As mentioned earlier, all additional activity is important for a person's health. From that perspective it is even more important that the environments where we act encourage physical activity. Several of the women mentioned that there were good bike lanes in the city that they could use to commute to work. However, the Swedish climate was seen as a barrier against physical activity, and these women needed more indoor possibilities to be physically active.

### 4.5. Lifestyle changes and empowerment

#### 4.5.1. How to make a change on the community or societal level

The overarching aim of Sweden's national public health policy is to “create societal conditions that will ensure good health, on equal terms, for the entire population” [25]. Public health interventions must be designed to reach individuals, groups, or the entire society, including all age groups, men and women, and migrants. In order to improve both the motivation for physical activity and the activity levels, the work must probably be spread on different arenas and based on different models. For a lifestyle change to be successful it needs to rely on earlier experiences and knowledge, be easy to incorporate into everyday life, and be perceived to solve some of one's problems and satisfy some of one's needs. If the goals were adjusted and more realistic, people would succeed more often [26]. The necessary support ought to be given by professionals, that is, educated training specialists, physiotherapists, public health workers, or dietitians if needed, who are able to provide reliable advice. Today, very many private training centers do not have educated staff and there might be a need for some new legislation to ensure that they do.

It is important to give support and advice to middle-aged migrant women about the beneficial effect of physical activity, and to increase the prescription of Physical Activity on Receipt by primary health caregivers, and it is also important that workplaces recommend, enable, and even subsidize participation in physical activity. The activities should focus on moderate physical activity, as well as on the teaching of relaxation methods, to be used before or after the physical activity in question – all this in order to increase heart health among middle-aged migrant women.

## 5. Conclusion

The study showed that the women from former Yugoslavia had decreased their level of physical activity after the move to Sweden. They had found it difficulties to find appropriate and realistic physical activities. Still, they desired to be more physically active. Further hindrances to physical activity and a healthy lifestyle were the harsh winter climate and lack of accessibility to indoor facilities. Furthermore, the women reported that they prioritized other activities, especially family and social life, above performing physical activity. Nevertheless, they reported as motivational factors, that physical activity could be a means to achieve better mental health as well as weight loss. Politicians ought to allocate funding, and public health worker could focus more on educational and empowerment interventions directed at this high risk group of immigrant women. They should also be informed about their increased risk of myocardial infarction. This, in order to stimulate increased physical activity among them, and it ought to be arranged in co-working with their own immigrant organization
